# Ammonium 4-(4-carb­oxy­phen­oxy)benzoate

**DOI:** 10.1107/S1600536810048841

**Published:** 2010-11-27

**Authors:** He-Ping Li, Seik Weng Ng

**Affiliations:** a Henan University of Traditional Chinese Medicine, Zhengzhou 450008, People’s Republic of China; b Department of Chemistry, University of Malaya, 50603 Kuala Lumpur, Malaysia

## Abstract

The anions of the title salt, NH_4_^+^·HO_2_CC_6_H_4_–O–C_6_H_4_CO_2_^−^, are linked by inter­molecular –CO_2_H⋯O_2_C– hydrogen bonds, forming a polyanionic chain in the crystal; adjacent chains are connected through the ammonium cation into a layer structure, with the ammonium cation serving as hydrogen-bond donor to four carboxyl­ate O atoms. The cation and anion both lie on special positions of 2 site symmetry. In the anion, the rings make a dihedral angle of 65.3 (1)°. The acid H atom is disordered about the special position.

## Related literature

For the crystal structures of two modifications of ­oxy-4,4′-bis­(benzoic acid), see: Dey & Desiraju (2005 ▶); Potts *et al.* (2007 ▶).
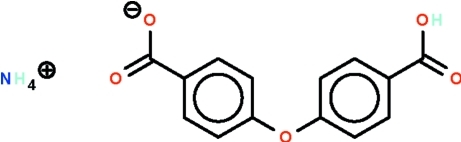


## Experimental

### 

#### Crystal data

NH_4_^+^·C_14_H_9_O_5_^−^
*M* *_r_* = 275.25
Orthorhombic, 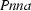

*a* = 6.1916 (1) Å
*b* = 28.5483 (6) Å
*c* = 7.1123 (1) Å
*V* = 1257.17 (4) Å^3^
*Z* = 4
Mo *K*α radiation
μ = 0.11 mm^−1^
*T* = 293 K
0.50 × 0.40 × 0.30 mm

#### Data collection

Bruker SMART APEX diffractometer
3444 measured reflections
1434 independent reflections
1279 reflections with *I* > 2σ(*I*)
*R*_int_ = 0.014

#### Refinement

*R*[*F* ^2^ > 2σ(*F* ^2^)] = 0.049
*wR*(*F* ^2^) = 0.146
*S* = 1.04
1434 reflections
102 parameters
6 restraints
H atoms treated by a mixture of independent and constrained refinement
Δρ_max_ = 0.30 e Å^−3^
Δρ_min_ = −0.42 e Å^−3^

### 

Data collection: *APEX2* (Bruker, 2007 ▶); cell refinement: *SAINT* (Bruker, 2007 ▶); data reduction: *SAINT*; program(s) used to solve structure: *SHELXS97* (Sheldrick, 2008 ▶); program(s) used to refine structure: *SHELXL97* (Sheldrick, 2008 ▶); molecular graphics: *X-SEED* (Barbour, 2001 ▶); software used to prepare material for publication: *publCIF* (Westrip, 2010 ▶).

## Supplementary Material

Crystal structure: contains datablocks global, I. DOI: 10.1107/S1600536810048841/hg2754sup1.cif

Structure factors: contains datablocks I. DOI: 10.1107/S1600536810048841/hg2754Isup2.hkl

Additional supplementary materials:  crystallographic information; 3D view; checkCIF report

## Figures and Tables

**Table 1:** Hydrogen-bond geometry (Å, °)

*D*—H⋯*A*	*D*—H	H⋯*A*	*D*⋯*A*	*D*—H⋯*A*
O1—H1⋯O1^i^	0.84 (1)	1.70 (3)	2.490 (2)	156 (6)
N1—H11⋯O1^i^	0.88 (1)	2.14 (1)	2.962 (2)	155 (1)
N1—H12⋯O2	0.88 (1)	2.10 (2)	2.827 (1)	139 (2)

Symmetry code: (i) 
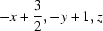
.

**Fig. 1.**
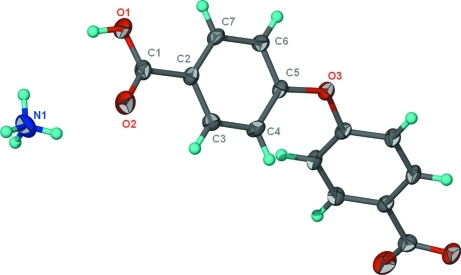
Thermal ellipsoid plot (Barbour, 2001) of [NH4]+ [HO2CC6H4–O–C6H4CO2]– at the 50% probability level. Hydrogen atoms are drawn as spheres of arbitrary radius.

**Fig. 2.**
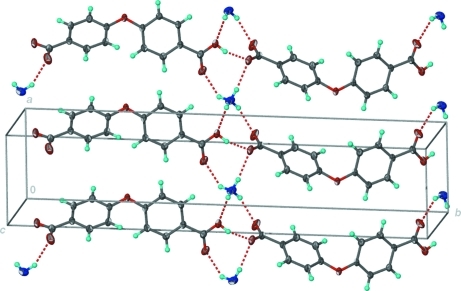
Layer structure projected onto the unit cell.

## supplementary crystallographic information

### Comment

We have been studying the co-crystals of carboxylic acids and amines. In the present study, the reaction of 4,4'-oxybis(benzoic acid) and tri-*n*-propylamine is expected to yield either the neutral co-crystal or the ammonium carboxylate. However, the amine has probably decomposed after being left in solution for several weeks. The product is ammonium hydrogen 4,4'-oxybis(benzoate) (Scheme I, Fig. 1). The non-hydrogen atoms of the benzoate portion of the anion nearly flat (r.m.s. deviation 0.10 Å); the two planes are aligned 65.3 (1) °. The anions are linked by an intermolecular –CO_2_H···O_2_C– hydrogen bond to form a polyanionic chain; adjacent chains are connected through the ammonium cation into a layer structure. The ammonium cation is hydrogen-bond donor to four carboxylate O atoms (Fig. 2). The cation and anion both lie on special positions of *2* site symmetry. The parent carboxylic acid itself crystallizes in two modifications (Dey & Desiraju, 2005; Potts *et al.*, 2007).

### Experimental

4,4'-Oxybis(benzoic acid) (0.25 mmol, 0.065 g) was dissolved in a water-ethanol (50 ml/100 ml *v*/*v*) mixture. Tri-*n*-propylamine (33% aqueous solution) was added until the solution registered a neutral pH. The mixture was then set aside for a several weeks; colorless crystals were isolated.

### Refinement

Carbon-bound H-atoms were placed in calculated positions (C–H 0.93 Å) and were included in the refinement in the riding model approximation, with *U*_iso_(H) set to 1.2*U*_eq_(C).

The acid and ammonium H-atoms were located in a difference Fourier map, and were refined with distance restraints of O–H 0.84±0.01 and N–H 0.88±0.01 Å. The temperature factor of the acid H atom was refined whereas that of the ammonium H atoms were tied by a factor of 1.2 times. For the ammonium H-atoms, because the N atom lies on a special position, the H···H distance was restrained to 1.43±0.01 Å.

### Crystal data

**Table:**

NH_4_^+^·C_14_H_9_O_5_^−^	*F*(000) = 576
*M**_r_* = 275.25	*D*_x_ = 1.454 Mg m^−^^3^
Orthorhombic, *P**n**n**a*	Mo *K*α radiation, λ = 0.71073 Å
Hall symbol: -P 2a 2bc	Cell parameters from 2311 reflections
*a* = 6.1916 (1) Å	θ = 2.9–27.6°
*b* = 28.5483 (6) Å	µ = 0.11 mm^−^^1^
*c* = 7.1123 (1) Å	*T* = 293 K
*V* = 1257.17 (4) Å^3^	Block, colorless
*Z* = 4	0.50 × 0.40 × 0.30 mm

### Data collection

**Table:**

Bruker SMART APEX diffractometer	1279 reflections with *I* > 2σ(*I*)
Radiation source: fine-focus sealed tube	*R*_int_ = 0.014
graphite	θ_max_ = 27.5°, θ_min_ = 2.9°
ω scans	*h* = −6→8
3444 measured reflections	*k* = −36→29
1434 independent reflections	*l* = −9→5

### Refinement

**Table:**

Refinement on *F*^2^	Primary atom site location: structure-invariant direct methods
Least-squares matrix: full	Secondary atom site location: difference Fourier map
*R*[*F*^2^ > 2σ(*F*^2^)] = 0.049	Hydrogen site location: inferred from neighbouring sites
*wR*(*F*^2^) = 0.146	H atoms treated by a mixture of independent and constrained refinement
*S* = 1.04	*w* = 1/[σ^2^(*F*_o_^2^) + (0.0922*P*)^2^ + 0.4317*P*] where *P* = (*F*_o_^2^ + 2*F*_c_^2^)/3
1434 reflections	(Δ/σ)_max_ = 0.001
102 parameters	Δρ_max_ = 0.30 e Å^−^^3^
6 restraints	Δρ_min_ = −0.42 e Å^−^^3^

### Fractional atomic coordinates and isotropic or equivalent isotropic displacement parameters (Å^2^)

**Table:**

	*x*	*y*	*z*	*U*_iso_*/*U*_eq_	Occ. (<1)
O1	0.83559 (19)	0.46053 (3)	0.15346 (17)	0.0429 (4)	
H1	0.748 (8)	0.4828 (15)	0.140 (6)	0.10 (2)*	0.50
O2	0.5981 (2)	0.43933 (4)	0.37136 (18)	0.0536 (4)	
O3	1.1215 (2)	0.2500	0.2500	0.0325 (4)	
C1	0.7477 (2)	0.43024 (5)	0.26451 (19)	0.0320 (3)	
C2	0.8424 (2)	0.38203 (4)	0.25651 (17)	0.0266 (3)	
C3	0.7341 (2)	0.34508 (5)	0.34202 (18)	0.0302 (3)	
H3	0.6034	0.3506	0.4026	0.036*	
C4	0.8184 (2)	0.30004 (5)	0.33813 (18)	0.0304 (3)	
H4	0.7450	0.2754	0.3950	0.036*	
C5	1.0136 (2)	0.29244 (4)	0.24811 (16)	0.0254 (3)	
C6	1.1244 (2)	0.32879 (5)	0.16242 (18)	0.0292 (3)	
H6	1.2555	0.3232	0.1027	0.035*	
C7	1.0374 (2)	0.37357 (4)	0.16673 (18)	0.0298 (3)	
H7	1.1105	0.3981	0.1089	0.036*	
N1	0.2500	0.5000	0.2884 (4)	0.0503 (5)	
H11	0.3506 (16)	0.5123 (6)	0.2161 (16)	0.060*	
H12	0.308 (3)	0.4771 (5)	0.355 (2)	0.060*	

### Atomic displacement parameters (Å^2^)

**Table:**

	*U*^11^	*U*^22^	*U*^33^	*U*^12^	*U*^13^	*U*^23^
O1	0.0450 (7)	0.0203 (5)	0.0633 (8)	0.0051 (4)	0.0098 (5)	0.0037 (4)
O2	0.0553 (8)	0.0374 (6)	0.0681 (8)	0.0191 (5)	0.0230 (6)	0.0050 (5)
O3	0.0327 (7)	0.0164 (6)	0.0485 (8)	0.000	0.000	−0.0001 (5)
C1	0.0339 (7)	0.0233 (6)	0.0387 (7)	0.0044 (5)	−0.0013 (5)	−0.0041 (5)
C2	0.0321 (7)	0.0194 (6)	0.0284 (6)	0.0019 (5)	−0.0005 (5)	−0.0016 (4)
C3	0.0309 (7)	0.0269 (7)	0.0329 (7)	0.0015 (5)	0.0058 (5)	−0.0014 (5)
C4	0.0366 (7)	0.0223 (6)	0.0323 (7)	−0.0030 (5)	0.0062 (5)	0.0024 (5)
C5	0.0328 (7)	0.0167 (6)	0.0266 (6)	0.0011 (4)	−0.0016 (5)	−0.0020 (4)
C6	0.0308 (7)	0.0219 (6)	0.0350 (7)	0.0006 (5)	0.0070 (5)	−0.0013 (5)
C7	0.0360 (8)	0.0183 (6)	0.0352 (7)	−0.0012 (5)	0.0062 (5)	0.0019 (4)
N1	0.0342 (10)	0.0516 (12)	0.0651 (13)	0.0082 (9)	0.000	0.000

### Geometric parameters (Å, °)

**Table:**

O1—C1	1.2914 (18)	C3—H3	0.9300
O1—H1	0.841 (10)	C4—C5	1.385 (2)
O2—C1	1.2260 (18)	C4—H4	0.9300
O3—C5^i^	1.3833 (13)	C5—C6	1.3852 (18)
O3—C5	1.3833 (13)	C6—C7	1.3876 (17)
C1—C2	1.4973 (17)	C6—H6	0.9300
C2—C7	1.3867 (19)	C7—H7	0.9300
C2—C3	1.3902 (18)	N1—H11	0.881 (7)
C3—C4	1.3880 (18)	N1—H12	0.882 (8)
			
C1—O1—H1	108 (4)	C5—C4—H4	120.6
C5^i^—O3—C5	122.29 (15)	C3—C4—H4	120.6
O2—C1—O1	123.76 (13)	O3—C5—C4	123.65 (11)
O2—C1—C2	120.94 (13)	O3—C5—C6	114.94 (12)
O1—C1—C2	115.30 (12)	C4—C5—C6	121.19 (11)
C7—C2—C3	119.32 (11)	C5—C6—C7	119.25 (12)
C7—C2—C1	121.23 (12)	C5—C6—H6	120.4
C3—C2—C1	119.45 (12)	C7—C6—H6	120.4
C4—C3—C2	120.83 (12)	C2—C7—C6	120.55 (12)
C4—C3—H3	119.6	C2—C7—H7	119.7
C2—C3—H3	119.6	C6—C7—H7	119.7
C5—C4—C3	118.86 (12)	H11—N1—H12	108.6 (10)
			
O2—C1—C2—C7	−166.60 (14)	C5^i^—O3—C5—C6	−151.57 (12)
O1—C1—C2—C7	12.97 (19)	C3—C4—C5—O3	174.06 (11)
O2—C1—C2—C3	12.8 (2)	C3—C4—C5—C6	−0.17 (19)
O1—C1—C2—C3	−167.60 (13)	O3—C5—C6—C7	−174.86 (10)
C7—C2—C3—C4	−0.1 (2)	C4—C5—C6—C7	−0.15 (19)
C1—C2—C3—C4	−179.57 (12)	C3—C2—C7—C6	−0.20 (19)
C2—C3—C4—C5	0.3 (2)	C1—C2—C7—C6	179.23 (12)
C5^i^—O3—C5—C4	33.86 (10)	C5—C6—C7—C2	0.3 (2)

Symmetry codes: (i) *x*, −*y*+1/2, −*z*+1/2.

### Hydrogen-bond geometry (Å, °)

**Table:**

*D*—H···*A*	*D*—H	H···*A*	*D*···*A*	*D*—H···*A*
O1—H1···O1^ii^	0.84 (1)	1.70 (3)	2.490 (2)	156 (6)
N1—H11···O1^ii^	0.88 (1)	2.14 (1)	2.962 (2)	155 (1)
N1—H12···O2	0.88 (1)	2.10 (2)	2.827 (1)	139 (2)

Symmetry codes: (ii) −*x*+3/2, −*y*+1, *z*.

## References

[bb1] Barbour, L. J. (2001). *J. Supramol. Chem.* **1**, 189–191.

[bb2] Bruker (2007). *APEX2* and *SAINT* Bruker AXS Inc., Madison, Wisconsin, USA.

[bb3] Dey, A. & Desiraju, G. R. (2005). *Chem. Commun.* pp. 2486–2488. 10.1039/b502516h 15886779

[bb4] Potts, S., Bredenkamp, M. W. & Gertenbach, J.-A. (2007). *Acta Cryst.* E**63**, o2887.

[bb5] Sheldrick, G. M. (2008). *Acta Cryst.* A**64**, 112–122. 10.1107/S0108767307043930 18156677

[bb6] Westrip, S. P. (2010). *J. Appl. Cryst.* **43**, 920–925.

